# Knowledge, attitudes and practices on malaria in relation to its transmission among primary school children in Bagamoyo district, Tanzania

**DOI:** 10.5281/zenodo.10785032

**Published:** 2016-02-19

**Authors:** Deborah Sumari, Angel Dillip, Vitalis Ndume, Joseph P. Mugasa, Paul S. Gwakisa

**Affiliations:** 1 School of Life Sciences and Bioengineering, The Nelson Mandela African Institution of Science and Technology, P.O. Box 447, Arusha, Tanzania.; 2 Ifakara Health Institute, P.O. Box 78373, Dares Salaam, Tanzania.; 3 Dares Salaam Institute of Technology, P. O. Box 2958, Dares Salaam, Tanzania.; 4 National Institute for Medical Research, Amani Medical Research Centre, P.O. Box 81, Muheza, Tanga, Tanzania.; 5 Genome Science Centre and Department of Veterinary Microbiology and Parasitology, Sokoine University of Agriculture, P.O. Box 3019, Morogoro, Tanzania.

## Abstract

**Background:**

Communities’ knowledge, attitudes and practices on malaria disease often remain unobserved during malaria control efforts. In Tanzania, many studies focus on increasing community knowledge and awareness on malaria prevention but the potential participation and contribution of schoolchildren towards knowledge, attitudes and practices on malaria has received little attention. We investigated the knowledge and understanding of primary school children on malaria transmission, recognition of symptoms, treatment seeking behaviour, preventive measures and practices in order to potentially include this group in Tanzania’s malaria control efforts.

**Materials and methods:**

125 children were recruited from three purposively selected primary schools in Bagamoyo district, Tanzania. A semi-structured interview guide, including both closed and open-ended questions, was used to collect information from the participants to obtain their knowledge and understanding on malaria transmission, treatment and prevention.

**Results:**

More than half of the school children (79/125; 63.2% ) had knowledge on malaria as a disease and its transmission; 101/125 (80.8%) of the respondents reported that going to the hospital was their immediate care-seeking behaviour once they felt malaria symptoms, while 14/125 (11.2%) opted for self-medication. With regard to malaria prevention and control, 115/125 (92.0%) of the respondents reported using bednets as their main malaria prevention strategy, while 6/125 (4.8%) preferred the use of medicine, mostly artemether lumefantrine, as prophylaxis. Narratives obtained were able to explain clearly the rationale behind different options children took to treat and to protect themselves against malaria.

**Conclusions:**

Findings indicated that primary school children in Bagamoyo district are aware of malaria, its symptoms and preventive measures, although some had misconceptions and could not associate the disease with its transmission. We conclude that inclusion of school children on malaria control educational programmes could yield substantial benefits towards malaria elimination.

## 1 Introduction

Malaria continues to pose a major public health burden in the United Republic of Tanzania, is the leading cause of in - and outpatient illness, and remains an impediment to socioeconomic growth and welfare, especially in rural areas [1]. A total of 93% of the Tanzanian population lives in areas where malaria is transmitted, whereas 60% of the malaria-endemic areas are characterised as having stable perennial transmission [2]. Malaria cases are reported to contribute to 42% of hospital diagnoses and 32% of reported hospital deaths over the last decade, with a higher prevalence in rural than in urban areas [3]. Despite the malaria burden, records from a malaria indicator survey show that malaria cases have been halved between 2008 and 2012 [4,5]. This decline is mostly due to the implementation and roll-out of malaria interventions, such as insecticide-treated nets (LLINs), indoor residual spraying (IRS) and the use of chemotherapies [6]. However, success of malaria control efforts is also dependent on the level of understanding, attitude and socio-cultural aspects of malaria prevention and treatment-seeking behaviours in the community [7].

A considerable number of reports on knowledge, attitude and practices relating to malaria and its control is available from different parts of Africa. Most of these reports concluded that misconceptions concerning malaria still exist and practices on malaria control are still unsatisfactory [8]. Furthermore, many studies have recommended more efforts to be directed towards controlling and improving malaria knowledge in the community [9]. Most studies concentrated on adults and only few [10,11] explored knowledge and perceptions among schoolchildren under the age of 14 years. Unlike adults, schoolchildren are more vulnerable to malaria infection, and about 50% of all malaria deaths in Africa occur in schoolchildren [12]. Studies conducted in Ethiopia [9,13] focused on peoples’ knowledge, attitude and practice on malaria in households, the population mean ages of which were 37.84 and 37.86 years, respectively. Other studies conducted in Swaziland [14], Mozambique [15] and Cameroon [16] also focused on adults’ knowledge, attitude and practice on malaria. In Tanzania, a recent study conducted in Rufiji [17] focused on measuring knowledge, attitude and behaviours towards malaria and malaria-like illnesses, and its participants’ ages ranged from 18-80 years. Another study conducted in Kilosa, eastern Tanzania [18], focused on understanding the knowledge and practice of community members and health workers on non-malaria fevers, in which the study populations were adults similar to a study conducted in rural northwest Tanzania [8].

The aim of this study was to investigate the level of knowledge, attitude and practice on malaria among primary schoolchildren aged 6 to 14 years, with a focus on malaria transmission, care-seeking behaviour and prevention in order to evaluate the potential of involving schoolchildren in malaria control strategies.

## 2 Materials and methods

### 2.1 Study area and population

**Figure 1.**
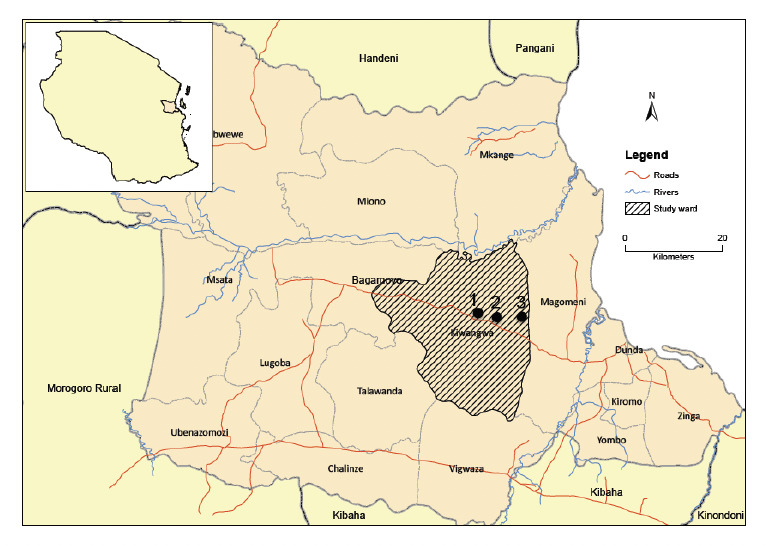
Bagamoyo map showing the study ward and study villages (1 = Mwavi, 2 = Fukayosi, 3 = Kidomole).

The study was conducted in Bagamoyo district in Pwani Region, Tanzania (Figure 1). The district is bordered by the Indian Ocean in the east, and Ruvu and Wami rivers in the west and north, respectively. In the south it is bordered by an uninhabited forest reserve. According to the National Census of 2012, Bagamoyo’s population is 311,740 [19], of which 81,000 are village inhabitants. The climate is hot in the months of November and December (average temperature 26°C), and cool in June (average temperature 19°C). The catchment area is primarily rural with low to moderate malaria transmission [4,5].

### 2.2 Study design and sampling

This was a mixed-method study. A semi-structured interview guide combining both closed and open-ended questions was used to collect information from schoolchildren. Three villages, Fukayosi, Mwavi and Kidomole, were selected based on slide positivity rates from microscopy records of symptomatic children attending dispensaries (unpublished data), and one primary school was selected from each village. Participants were purposively selected by recruiting children aged 6-14 years as described previously [20]. The sample size was calculated using a standard formula for prevalence studies [21], as follows:

n=Z2P(1−P)d2

Where *n* is sample size, Z is a *Z* statistic value of 1.96 at confidence level of 95%. *P* is considered prevalence at 9% [5] at 95% confidence interval and *d* is a 5% relative precision. To account for dropout from school during the study, 10% of the calculated sample size was added. The participants were selected from all classes in the schools (classes one to seven) in order to capture age diversity.

### 2.3 Data collection

Prior to data collection, a semi-structured interview guide was developed and pre-tested for validity. As indicated above, the guide included both closed and open-ended questions examining and exploring the general understanding on malaria disease, its transmission, treatment and preventive measures. The interview guide was developed in English and later translated into Kiswahili. The interview was conducted by two trained research assistants under supervision of the lead researcher.

In order to collect adequate and correct information, sensitisation and mobilisation meetings involving researchers, schoolteachers, children and parents were conducted, in which the study objectives were explained. The aim of such meetings was to inform the children and parents about the importance for them to share knowledge on malaria. Children whose parents or guardians consented were requested to sign assent forms before being enrolled in the study. A total of 125 pupils from Mwavi (*n*=26), Fukayosi (*n*=62) and Kidomole (*n*=37) were recruited.

### 2.4 Ethical considerations

Ethical approval was obtained from the Institutional Review Board (No. IHI/IRB/No: 34-2013) of the Ifakara Health Institute and the Medical Research Coordinating Committee of the National Institute for Medical Research (No. NIMR/HQ/R.8a/Vol. IX/1705). Informed consent forms (ICFs) were distributed to the pupils by teachers and researchers for presenting to parents and/or guardians one week prior to data collection. The ICFs explained risks, benefits and confidentiality associated with the children’s participation in the study. In case a parent or guardian was illiterate, schoolteachers provided assistance to acquaint such parents/guardians with the ICF contents. Additionally, the confidentiality of all participants was assured by using identity study numbers. Furthermore, permission to conduct this study was granted by regional and district authorities in the study area.

### 2.5 Data management and analysis

For quantitative data, descriptive statistics were carried out to measure relative frequencies and percentages of variables. Narratives from qualitative data were coded and managed using a framework of themes that addresses key issues as per study objectives. Coding and coding consistency checking was carried out. A list of codes was developed, reviewed and grouped into themes and categories for analysis. The following main three themes formed the focus of both findings: malaria symptoms, care-seeking behaviour and prevention.

## 3 Results

### 3.1 Socio-demographic characteristics of the study participants

Seventy-eight (62.4%) girls and 47 (36.7%) boys were enrolled in the study. Fifteen children (12%) were 6-8 years, 54 (43.2%) were 9-11 years, and 56 (44.8%) were 12-14 years of age. In addition, distribution of the children by school classes was rather even, with 60 (48%) recruited from standards I-III and 65 (52%) from standards IV-VI (Table 1).

**Table 1.** Socio-demographic characteristics of study participants (*n*=125).

Variable	Frequency (%)
**Gender**	
Girl	78 (62.4)
Boy	47 (37.6)
**Age (yrs)**	
6-8	15 (12.0)
9-11	54 (43.2)
12-14	56 (44.8)
**Class (standard)**	
I-III	60 (48.0)
IV-VI	65 (52.0)

### 3.2 Knowledge on malaria

The schoolchildren from the three villages had similar levels of understanding on malaria and its transmission. More than half (79/125; 63.2%) had proper knowledge of malaria as a disease and its transmission. Essentially, the study participants were aware that malaria is transmitted by a mosquito bite and mentioned that not all mosquitoes can transmit malaria except female mosquitoes called *Anopheles*. Conversely, few respondents (10/125; 8%) did not understand the type of mosquitoes that transmit malaria (Table 2). However, some participants (7/125; 5.6%) had misconceptions about the mode of malaria transmission and associated it with eating dirty foods, contact with a malaria patient or going to the toilet without shoes. The following narratives indicate how participants explained their knowledge on malaria disease:

**Table 2.** Knowledge on malaria disease, transmission, care-seeking behaviour and prevention (*n*=125) .

Variable	Frequency (%)
**Knowledge about the disease**	
Mosquito-transmitted disease	79 (63.2)
A disease/others	29 (23.2)
Misconception (eating dirty food, con-tacting a malaria patient, etc.)	7 (5.6)
‘Don’t know’	10 (8.0)
**Malaria symptoms**	
Fever and headache	70 (56.0)
Vomiting and diarrhoea	35 (28.0)
Other symptoms	11 (8.8)
‘Don’t know’	9 (7.2)
**Care-seeking behaviour**	
Go to hospital	101 (80.8)
Take medicine/others	19 (15.2)
‘Don’t know’	5 (4.0)
**Prevention**	
Use mosquito net	115 (92.0)
Other measures	6 (4.8)
‘Don’t know’	4 (3.2)
**Frequency of contracting malaria**	
Not had malaria for years	78 (62.4)
Once per month	35 (28.0)
Once per year	9 (7.2)
‘Don’t know’	3 (2.4)

*‘Malaria is a disease caused by a mosquito; transmitted by a female mosquito’ [child, 10 yrs].*

*‘Malaria is a disease causing body pain and fever; transmitted by being bitten by a mosquito which has malaria parasites’ [child, 11 yrs].*

*‘Malaria is a disease caused by a bite from a female mosquito’ [child, 12 yrs].*

*‘Malaria is a disease transmitted by eating dirty foods, you can also get malaria when you go to the toilet without shoes’ [child, 8 yrs].*

#### 3.2.1 Malaria symptoms

A high percentage of participants (105/125; 84%) had knowledge on malaria symptoms. Fever and headache were the most commonly mentioned symptoms (Table 2), as illustrated below by study participants:

*‘Sometimes I feel weak, dizzy, feverish, headache and feel like vomiting, I tell my mother to take me to the hospital for examination’ [child, 11 yrs].*

*‘Malaria is a disease caused by a mosquito bite, which causes body fatigue, fever, headache, body weakness and loss of appetite’ [child, 12 yrs].*

*‘When I feel headache, abdominal pain, fever and vomiting, I go to the dispensary for malaria check up’ [child, 13 yrs].*

#### 3.2.2 Care-seeking behaviour

When the participants were asked about treatment they take when sick, most of them (101/125; 80.8%) reported that when experiencing malaria symptoms, they normally ask their parents/guardians to take them to a health facili-ty/hospital. However, about 19 (15.2%) acknowledged that once they feel sick their parents/guardians would simply buy antimalarial drugs from medical stores. The same is supported by qualitative information, which went further into looking at the rationale for choosing the mentioned resort, as narrated below:

*‘When I feel feverish, I know that it is malaria and I immediately tell my mother and we go to the hospital for check up’ [child, 7 yrs].*

*‘My mother takes me to hospital immediately when we suspect malaria, at the hospital there are modern facilities to test malaria [child, 11 yrs].*

*‘They buy ALU from a drug store and give it to me immediately when I don’t feel good, the drug store is not very far from where we live’ [child, 10 yrs].*

It was also revealed that children with knowledge about antimalarial drugs could further describe colours of the drugs and name the antimalarial drugs they always take:

*‘When I get malaria, I go to the hospital, they give me yellow medicines (ALU) and paracetamol’ [child, 9 yrs].*

*‘I tell my parent to give me ALU when I feel malaria symptoms’ [child, 10 yrs].*

#### 3.2.3 Malaria prevention

Our study found that 115/125 (92%) of the respondents knew about malaria-preventive measures and reported to use bednets as their primary measure for protection against malaria (Table 2). Few respondents (6/125; 4.8%) reported to have taken medicine, mostly ALU, for malaria prevention, and cleaning home environments to prevent mosquitoes from breeding. Likewise, the respondents could also describe how their family members could get malaria by not taking proper preventive measures:

*‘If you use a mosquito net, you will not get malaria frequently, as for me I sleep under bed net everyday’ [child, 12 yrs].*

*‘My grandmother got malaria once because she works hard and doesn't put her mosquito net properly before going to bed therefore mosquitoes bite her’ [child, 7 yrs].*

*‘I like to use a mosquito net every time I go to bed and clean our home environment to prevent malaria causing mosquitoes’ [child, 12 yrs].*

#### 3.2.4 Frequency of contracting malaria

Our findings show that 78 (62.4%) of the study participants reported not to contract malaria frequently or for years (Table 2), whereas 35 (28%) and 9 (7.2%) reported to contract malaria once per month and per year, respectively. However, 3 (2.4%) of the respondents could not mention how often they contracted malaria.

*‘I get malaria almost twice a year and I think because our mosquito net has several holes where mosquitoes get in and bite me’ [child, 10 yrs]*.

*‘I rarely get sick but I remember two years ago when I went to fetch water at Lamboni area I got malaria’ [child, 11 yrs].*

The respondents went further and reported about family members in the household who frequently suffered from malaria. They indicated that the frequency of malaria bouts could have been caused by not using a mosquito net and bad sleeping habits:

*‘My young brother gets malaria I think every month because he doesn’t like sleeping in a mosquito net’ [child, 11 yrs]*.

*‘My young sister gets malaria often because she usually rolls on the bed and her body touches the mosquito net where the mosquitoes bite her’ [child, 11 years].*

## 4 Discussion

We investigated knowledge, attitudes and practice of primary schoolchildren regarding malaria transmission, prevention and control. A number of studies have been conducted to assess knowledge, attitudes and practices on malaria, but very few have been conducted on primary schoolchildren, especially in Tanzania. The current study used primary schoolchildren aged 6-14 years. Information was captured from a diverse age group of children purposively selected from three rural villages. However, we recognise some limitations. Many parents/guardians were not ready to consent for their children to participate into the study despite the fact that their children were ready to assent. In this regard, more information could have been captured from many more children. Health knowledge among community members along the coast is generally scarce, and some individuals might have unrealistic beliefs associated with their children’s health. It is also possible that parental refusals to allow their children to participate in the study were influenced by misconceptions that were caused by previous research undertakings.

Our findings indicate that the majority of primary schoolchildren in rural Bagamoyo have knowledge on malaria transmission, causes, symptoms, treatment and preventive measures. The children were able to describe malaria as a disease transmitted by infected female mosquitoes. This observation further supports findings of another study conducted in southwestern Tanzania [10], which reported that more than 80% of school children had correct knowledge on malaria transmission. Other studies conducted in Ethiopia [22] and Cameroon [11] also showed high percentages of children who were aware of malaria symptoms and causes. Contrary to our findings, studies with adults conducted in Tanzania [23], Nigeria [24] and Zimbabwe [25] reported low knowledge and awareness about causes of malaria and prevention in communities.

We suggest that the level of malaria knowledge among the schoolchildren is due to national and local public awareness programmes through mass media such as radio and television. However, there were misconceptions related to malaria transmission among some schoolchildren. Some of the cited misconceptions include going to the toilet without shoes, contact with malaria patients and eating dirty foods, which had also been reported previously in Tanzania, India and Ethiopia [10,22,26,27]. Moreover, a small proportion (8%) of the school children had no knowledge of malaria transmission. This study emphasises that further attention is needed towards the content and kind of malaria messages that are delivered in the community. Although more than half of the children were knowledgeable about malaria and its transmission, we conclude that there is still a lack of proper information about malaria that we report as has been found in studies involving adults [8, 28].

Knowledge on signs and symptoms associated with malaria was tested, and more than half (56%) of the schoolchildren mentioned fever and headache as common symptoms associated with malaria. Some children (28%) further associated vomiting and diarrhoea with malaria, while others mentioned loss of appetite, dizziness and shivering. These findings corroborate those from previous studies [24,29,30].

Knowledge on care-seeking behaviour was well captured. The majority of children mentioned that hospitals and dispensaries are their immediate places for seeking malaria treatment, while 15.2% mentioned that their parents opted for self-treatment. Although practices on self-treatment have also been a concern in the community [30-32], this study emphasises that self-treatment without a doctor’s prescription, could lead to disease complications and the possible emergence of drug resistance [33,34]. We accentuate that knowledge on malaria treatment should be given to the community, including schoolchildren, to avoid negative effects of self-treatment and health-related consequences.

A substantial proportion of the children (92%) reported that use of bednets was a primary preventive method of malaria. This observation is in agreement with the National Malaria report, which indicates that ~95% of Tanzanian communities possess mosquito nets and that 68% of the households use ITNs [5,32,35]. Deployment of ITNs and malaria intervention campaigns in the coastal area, Zanzibar and Bagamoyo indicated positive results on community awareness on malaria prevention, which resulted in a dramatic reduction in malaria prevalence [36,37]. Nevertheless, more knowledge should be given to the community and schoolchildren on the difference between ITNs and other mosquito nets for effective malaria prevention and control [38]. Therefore, the knowledge on malaria symptoms, transmission and practice shown by the schoolchildren in this study strongly warrants and supports their inclusion in educational programmes to further improve malaria control efforts.

## 5 Conclusions

This study shed light on the level of knowledge, attitudes and practices of schoolchildren on malaria in the Bagamoyo district, Tanzania. The schoolchildren indicated that they have knowledge of malaria, its symptoms and preventive measures. However, there were some misconceptions, as shown by some children on the mode of malaria transmission, which needs to be addressed. The majority of the children emphasized that attending hospitals and dispensaries are their primary care-seeking behaviour when contracting malaria, although some demonstrated that self-treatment is still common among the community. Therefore, the need for correct awareness to the community about self-treatment drawbacks is highly recommended in order to improve malaria control strategies and meet the malaria elimination goals.
